# Supplementary material for: Implementation of a lung cancer screening initiative in HIV-infected subjects
Source: PLoS One. 2021 Dec 10;16(12):e0260069. doi: 10.1371/journal.pone.0260069 (PMC8664191; doi:10.1371/journal.pone.0260069)
Supplement: Attachment — Submitted filename: Response to Reviewer.docx [file pone.0260069.s003.docx]

**Response to Reviewers**

**Title: Implementation of a lung cancer screening initiative in HIV-infected subjects**

The authors would like to thank the Reviewers and Editors for their careful review of our manuscript, and for providing us with their very helpful comments and suggestions to improve the quality of the manuscript. The following responses have been prepared to address all of the referees’ comments in a point-by-point fashion.

Journal Requirements:

**Please review your reference list to ensure that it is complete and correct. If you have cited papers that have been retracted, please include the rationale for doing so in the manuscript text, or remove these references and replace them with relevant current references. Any changes to the reference list should be mentioned in the rebuttal letter that accompanies your revised manuscript. If you need to cite a retracted article, indicate the article’s retracted status in the References list and also include a citation and full reference for the retraction notice.**

We have reviewed our reference list and we havent been able to find any anomaly related to the issues highlighted.

**Comments to the Author**

1. If the authors have adequately addressed your comments raised in a previous round of review and you feel that this manuscript is now acceptable for publication, you may indicate that here to bypass the “Comments to the Author” section, enter your conflict of interest statement in the “Confidential to Editor” section, and submit your "Accept" recommendation.

Reviewer #1: All comments have been addressed

2. Is the manuscript technically sound, and do the data support the conclusions?

The manuscript must describe a technically sound piece of scientific research with data that supports the conclusions. Experiments must have been conducted rigorously, with appropriate controls, replication, and sample sizes. The conclusions must be drawn appropriately based on the data presented.

Reviewer #1: Yes

3. Has the statistical analysis been performed appropriately and rigorously?

Reviewer #1: Yes

4. Have the authors made all data underlying the findings in their manuscript fully available?

The [PLOS Data policy](http://www.plosone.org/static/policies.action#sharing) requires authors to make all data underlying the findings described in their manuscript fully available without restriction, with rare exception (please refer to the Data Availability Statement in the manuscript PDF file). The data should be provided as part of the manuscript or its supporting information, or deposited to a public repository. For example, in addition to summary statistics, the data points behind means, medians and variance measures should be available. If there are restrictions on publicly sharing data—e.g. participant privacy or use of data from a third party—those must be specified.

Reviewer #1: Yes

5. Is the manuscript presented in an intelligible fashion and written in standard English?

PLOS ONE does not copyedit accepted manuscripts, so the language in submitted articles must be clear, correct, and unambiguous. Any typographical or grammatical errors should be corrected at revision, so please note any specific errors here.

Reviewer #1: Yes

6. Review Comments to the Author

**Reviewer #1:**

This manuscript is greatly improved. All of my comments have been addressed. Thank you for your thorough revisions. I have a few remaining small edits:

**- Table 1. The IQR is missing for Pack years.**

We have included IQR in table 1.

**- On line 148, I think a word is missing. I think it should read: “…2.6% of participants were diagnosed with LC.”**

We have included the error, the word LC has added to the paper.

**- On line 189 I think that should read “pack-years” not “packs-year”.**

We have checked this error and we have changes the term “pack-years” for packs-year”. We have also changed “PACKS-YEAR” in table 1 into “PACK-YEARS”.

**We have spotted some minor errors in the paper after a thorough recheck. Below we show these changes we have made as long as you accordant with them.**

- On line 24 and 34, LC has been added in order to explain the meaning of the acronym LC (which in our article means lung cancer)

- On line 42, 44, 46, 123 and 190 the words “lung cancer” has been replaced by the acronym “LC”

- On line 45 and 179 “– “has been changed by a “,”.

- On line 47, the word “cancer” was missing after lung and before screening so we have added LC to address the issue that screening program is for lung cancer

- On line 155 the word “a” was a mistake and it was meant to be “as” so it has been changed.

- On line 193, “pack-year” has been changed for “pack-years”.
